# Does preoperative physical therapy/prehabilitation affect outcome or complications after surgery for lumbar disc herniation? A systematic review

**DOI:** 10.1016/j.bas.2025.104386

**Published:** 2025-08-06

**Authors:** João Pedro Oliveira, Mariana Casqueiro, João Paulo Andrade, Carla Reizinho

**Affiliations:** a Neurosurgery Department, Hospital de Egas Moniz, Unidade Local de Saúde Lisboa Ocidental, Portugal; b Department, Hospital da Luz, Lisbon, Portugal

**Keywords:** Rehabilitation, Pre-operative physical therapy, Prehabilitation, Lumbar disc herniation, Systematic review

## Abstract

**Introduction:**

Preoperative physical and psychological conditioning or ‘prehabilitation’, has emerged as a potential strategy to enhance surgical outcomes. While recent studies have investigated the role prehabilitation in spinal surgery, its specific role in lumbar disc herniation remains insufficiently defined.

**Objective:**

To evaluate the impact of preoperative physical therapy/rehabilitation on postoperative outcomes and complications in patients undergoing surgery for lumbar disc herniation, compared to standard preoperative care. before surgery for lumbar disc herniation in the global outcome compared with the preoperative usual care in waiting list.

**Methods:**

This systematic review searched 4 databases from January 2000 to March 2023. All studies (randomized clinical trials and observational studies) assessing the effects of prehabilitation in adult patients undergoing lumbar spine surgery, were eligible for inclusion. Five studies (n = 736) met inclusion criteria.

**Results:**

All included studies reported short-term improvements in pain, functional outcomes, and psychological readiness following prehabilitation. However, these benefits were not sustained at 6- or 12-months follow-up in most studies. Outcomes converged between intervention and control groups over time. One study highlighted that higher-intensity, supervised programs yielded greater early benefits than unsupervised protocols.

**Conclusion:**

Prehabilitation appears to be a promising intervention for enhancing short-term recovery following lumbar spine surgery. Nevertheless, its long-term effectiveness remains uncertain. The current evidence is limited by clinical heterogeneity and lack of lumbar disc herniation specific trials. Future long-term, standardized, high-quality studies are essential to define its role in surgical outcomes.

## Highlights

• Impact of Preoperative Rehabilitation on Lumbar Disc Herniation (LDH) Surgery Outcomes.
• Five studies with 736 participants, sourced from four databases, examining the effects of prehabilitation on LDH surgery outcomes.
• Short-term improvements in pain and functional measures were observed post-intervention, though these benefits did not persist in long-term follow-ups (6 months–1 year).
• The article underscores the necessity for future studies to explore long-term effects, standardized outcome measures, and the cost-effectiveness of prehabilitation in the context of LDH surgery.

## 1 Introduction

Low back pain (LBP) and radicular leg pain resulting from lumbar disc herniation (LDH) is among the most prevalent complaints in neurosurgery clinic, often leading to a debilitating condition that impairs normal daily activities. This condition represents a significant financial burden on the healthcare system, with a lifetime prevalence reaching as high as 84 % (Balagué et al., 2012) (see Table 1).

**Table 1:** Characteristics of included studies.

Study	Design	Population (n)	Intervention	Comparator	Outcomes Measured	Follow-up
1. Lindbäck et al., 2018	**RCT**	197	9-week supervised PT	Usual care	Pain (VAS), ODI, EQ-5D, FABQ-PA	**12** months
2. Fors et al., 2019	**RCT**	195	9-week supervised PT	Usual care	Strength, walking, FABQ-PA	**12** months
3. Marchand AA et al., 2021	**RCT**	60	6-week home exercise	Usual care	Pain (VAS), ODI	**6** months
4. Carrignan et al., 2020	Observational	234	PT + pharmacological therapy	No PT	Pain, EQ-5D	**12** months
5. Nielsenet PR et al., 2010	**RCT**	50	8-week prehab + early rehab	Standard care	Pain, HR-QoL, performance tests	**12** months

Conservative treatment should be considered as the initial approach for managing LDH in the absence of cauda equina syndrome or significant motor and neurological deficits (Yaman et al., 2024). It generally includes a combination of pain-relief medications (nonsteroidal anti-inflammatory drugs (NSAIDs), with opioids being reserved as a last resort) with physical therapy. Surgical decompression is recommended when patients continue to experience disabling pain and radiculopathy after sufficient rehabilitation, and imaging reveals compressive pathology (Lindbäck et al., 2018). In fact, there has been an increase in the number of patients undergoing spinal surgery over the last decades (Nielsen et al., 2010). The process of recovery following surgery can be challenging with 10 %–40 % of patients experiencing less than optimal outcomes, including persistent pain, the need for reoperation, reduced mobility, and varying levels of disability (Yorimitsu et al., 2001).

It is recognized that enhancing overall health before surgery is linked to improved postoperative outcomes and reduced complications for patients undergoing various types of surgical procedures (Nielsen et al., 2010). Indeed, in the time preceding surgery there seems to be an enhanced receptivity to changes in health behaviors, offering a strategic opportunity to introduce interventions aimed at enhancing physical well-being (Fors et al., 2019).

Prehabilitation, encompassing physical therapy and cognitive-behavioral therapy, seeks to boost patients' functional abilities before surgery by enhancing their physical fitness and their perception of pain, the surgical experience, and its outcomes (Parrish et al., 2021).

In this setting, recent studies have explored the role of preoperative rehabilitation for patients undergoing spinal surgery, although prehabilitation in lumbar disc herniation is yet to be defined.

## 2 Methods

### 2.1 Review protocol

The conduct and reporting of this review followed the up-to-date version of the Meta-analysis (PRISMA) 2020 Checklist (Page et al., 2021), as recommended by the Cochrane Handbook for Systematic Reviews of Interventions.

### 2.2 Search and eligibility criteria

We performed a specific search of English-language literature across 4 electronic databases: PubMed (MEDLINE), clinicaltrials.gov, Cochrane Library and Google Scholar to identify relevant studies until March 2023. We searched these databases using key words such as: lumbar disc herniation AND rehabilitation OR lumbar disc herniation AND pre-operative rehabilitation. The nature of the search was chosen to be as inclusive as possible ensuring inclusion of potentially relevant studies. All retrieved titles and abstracts were reviewed independently by 2 investigators (JPO and MC).

### 2.3 Inclusion and exclusion criteria

We included articles that were (1) in English language, (2) included adults only (18 years old or above), (3) published until March 2023, (4) randomized control trials (RCTs) or observational studies, containing information related to (5) lumbar disc herniation and rehabilitation [1 RCT] or (6) lumbar disc herniation and pre-operative rehabilitation [3 RCTs and 1 observational study]. We excluded RCTs and/or protocols with no published results and non-human studies.

### 2.4 Study selection and data extraction

**Fig. 1:**
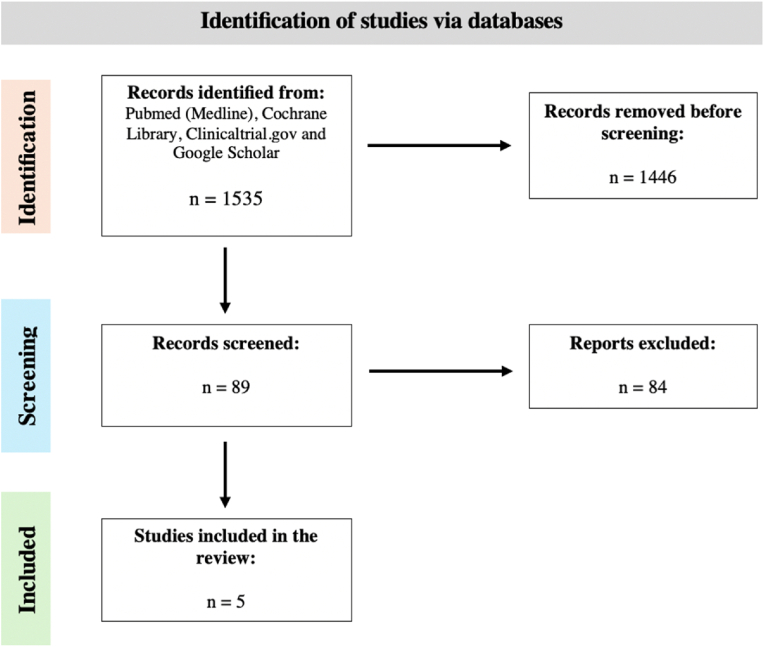
Flow chart identification of studies for this review.

Considering the entire focus across the four databases (PubMed/Medline, clinicaltrials.gov, Cochrane and Google Scholar), the initial search yielded 1535 potentially relevant studies. From this initial group, 1446 were removed before screening since they were clinical trials or protocols with no published results. The remaining 89 studies of these concerning the pre-operative rehabilitation in spinal lumbar surgery were screened based on eligibility using the inclusion criteria, resulting in 5 for which data were retrieved (Fig. 1).

### 2.5 Data synthesis

Given the clinical and methodological heterogeneity across included studies - including differences in patient populations, intervention types, outcome measures, and follow-up durations - a meta-analysis was not feasible. Therefore, we conducted a narrative synthesis structured around keys outcomes: pain symptoms, functional status, psychological readiness, and quality of life. Study results were grouped and compared based on outcome domains, direction and magnitude of effects, follow-up periods and intervention characteristics (e.g., supervised vs. unsupervised, duration). This approach facilitated the identification of patterns and divergences in reported findings.

## 3 Results

### 3.1 Study selection

A total of one thousand, five hundred and thirty-five (1535) literatures were found after searching Cochrane Library, PubMed, clinicaltrials.gov and Google Scholar, from which 1446 articles were initially removed as they did not have published results regarding the pre-operative rehabilitation in spinal lumbar surgery. As outlined in Fig. 1, upon eligibility screening 89 publications were assessed and 84 publications were excluded (reasons for exclusion: systematic reviews and meta-analysis, literature reviews and participants characteristics unmet).

### 3.2 Study characteristics

The mean age of participants across included studies was 54,9 years old, and 46,6 % were female.

Participants underwent a lumbar spinal procedure mainly because of lumbar spinal stenosis, lumbar disc herniation and spondylolisthesis, receiving various surgical modalities including laminectomy, laminectomy with fusion, discectomy, arthrodesis or no surgical intervention. Two rehabilitation interventions were identified: physical therapy (4 studies, (Lindbäck et al., 2018), (Fors et al., 2019), (Marchand et al., 2021), (Nielsen et al., 2010)) and physical therapy/pharmacological therapy (1 study (Carrignan et al., 2020),). In the different studies analyzed, physical therapy comprised various modalities namely muscle strength training, postural re-education, cardiovascular conditioning. The duration of rehabilitation interventions varied: a) 6 weeks (1 study (Marchand et al., 2021),); b) 8 weeks (1 study, (Nielsen et al., 2010); c) 9 weeks (2 studies, (Lindbäck et al., 2018), (Fors et al., 2019)) and d) 12 months (1 study (Carrignan et al., 2020),). Main outcomes included back pain intensity (5 studies, (Lindbäck et al., 2018), (Fors et al., 2019), (Marchand et al., 2021), (Carrignan et al., 2020), (Nielsen et al., 2010)), leg pain intensity (4 studies, (Lindbäck et al., 2018), (Fors et al., 2019), (Marchand et al., 2021), (Nielsen et al., 2010)), functional outcome (5 studies, (Lindbäck et al., 2018), (Fors et al., 2019), (Marchand et al., 2021), (Carrignan et al., 2020), (Nielsen et al., 2010)) and quality of life (4 studies, (Lindbäck et al., 2018), (Fors et al., 2019), (Carrignan et al., 2020), (Nielsen et al., 2010)). The studies were clinically heterogeneous, including both RCTs and observational studies.

### 3.3 Risk of bias assessment

**Fig. 2:**
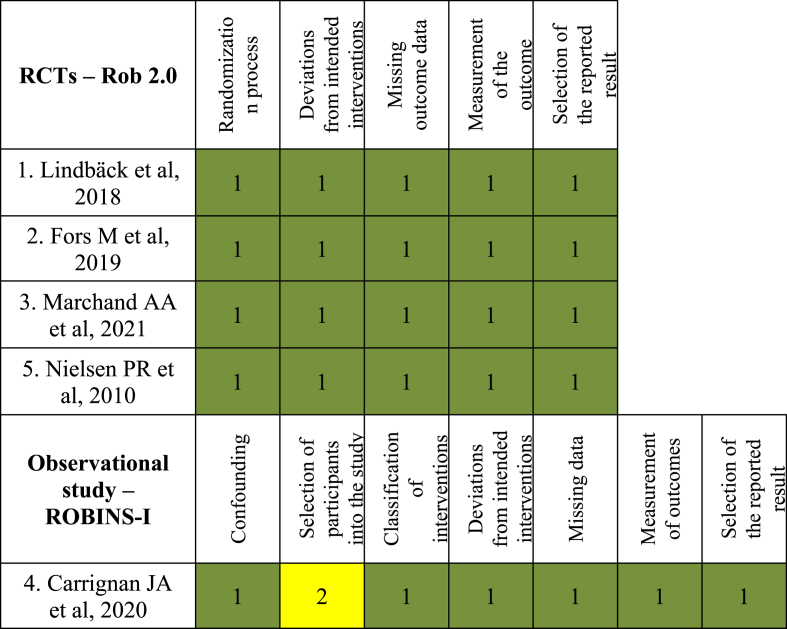
Schematic representation of risk of bias assessment.

The assessment for the risk of bias in included studies is demonstrated in Fig. 2, using Cochrane Risk Bias 2.0 (RoB 2.0) for RCTs, while observational studies require ROBINS-I (Risk of Bias in Non-randomized studies – of interventions). Each domain of bias was evaluated and color-coded: green indicates low risk (1), yellow indicates moderate risk (2). The majority of randomized controlled trials demonstrated low risk of bias across all domains, while the single observational study exhibited a moderate risk of bias primarily due to confounding and participant selection.

### 3.4 Main outcomes

#### 3.4.1 Symptomatic/clinical outcomes

Symptomatic/clinical outcomes comprised 2 domains, back and leg pain, mainly assessed by using the visual analog scale (VAS) for pain (4 studies, (Lindbäck et al., 2018), (Fors et al., 2019), (Marchand et al., 2021), (Carrignan et al., 2020)) and the modified Brief Inventory Questionnaire (1 study (Nielsen et al., 2010),). Overall, **prehabilitation appears effective in reducing pain symptoms in the short-term post-operative**, with short-term reductions in pain ((Lindbäck et al., 2018), (Fors et al., 2019), (Marchand et al., 2021), (Carrignan et al., 2020), (Nielsen et al., 2010)), and aligning with improved mobility (Carrignan et al., 2020)). VAS scores were significantly reduced following intervention, **with diminished effect sizes beyond 6–12 months,** with differences fading by 6 months (Marchand et al., 2021) or 12 months (Lindbäck et al., 2018), (Fors et al., 2019), (Carrignan et al., 2020), (Nielsen et al., 2010)), where intervention and control groups converged.

#### 3.4.2 Functional outcome

Functional outcome measured as quality of life was reported in all the five included studies. The Oswestry Disability Index (ODI) Score (4 studies, (Lindbäck et al., 2018), (Fors et al., 2019), (Marchand et al., 2021), (Carrignan et al., 2020)), the European Quality of Life-5D (EQ-5D) (2 studies, (Lindbäck et al., 2018), (Fors et al., 2019)), the Health-Related Quality of Life (HR-QoL) and the Roland Morris Questionnaire (1 study (Nielsen et al., 2010),) were the tools applied in this review to measure this type of outcome. Taken together, **functional improvements are consistently evident following prehabilitation but tend to converge with control groups at 1-year follow-up.**

#### 3.4.3 Other outcomes

Among the five analyzed studies, besides the main outcomes (symptomatic and functional) there were several secondary outcomes which varied, not being present in all of them. Fear-Avoidance Beliefs Physical Activity (FABQ-PA) (2 studies, (Lindbäck et al., 2018), (Fors et al., 2019)), revealing significant early decreases in fear-avoidance beliefs post-intervention, which may contribute to improved mobility; Performance-based outcomes (timed up-and-go and sit-to-stand) (1 study (Nielsen et al., 2010)) reflecting improvements in mobility and lower extremity strength. These measures support the idea that **psychological readiness and physical performance are positively influenced by prehabilitation in short-term.**

## 4 Discussion

### 4.1 Summary of main results

This systemic review included five studies with a total of 736 adult participants undergoing lumbar spinal surgery (for disc herniation, spinal stenosis, spondylolisthesis or degenerative disc disease). Preoperative rehabilitation was associated with **short-term improvements** in back and leg pain, physical function, mobility and psychological readiness ((Lindbäck et al., 2018), (Fors et al., 2019), (Marchand et al., 2021), (Carrignan et al., 2020), (Nielsen et al., 2010)). The PREPARE trial and its secondary analysis ((Lindbäck et al., 2018), (Fors et al., 2019)), showed early gains in leg strength, walking ability, and fear-avoidance beliefs. **Importantly, however, these differences were not sustained at 6 month follow up** (Marchand et al., 2021) **and at 12 month follow up** (Lindbäck et al., 2018)**,** (Fors et al., 2019)**,** (Carrignan et al., 2020)**,** (Nielsen et al., 2010) both groups' outcomes were leveled out. Only one study (Lindbäck et al., 2018) reported a modest persistent difference in physical activity only for the rehabilitation group. The intensity and supervision of rehabilitation program (as noted by Carrignan et al. (2020)) may influence the magnitude and duration of benefit.

Altogether, the evidence supports **prehabilitation as a value strategy for enhancing short-term postoperative recovery**. However, there is a pressing needed for high-quality, diagnosis-specific clinical trials with extended follow-up periods to elucidate how theses interventions can be optimized and sustained.

### 4.2 Limitations

Firstly, the primary limitation of this systemic review is the **heterogeneity in study populations**, as most included trials focused on mixed degenerative lumbar conditions – not exclusively on lumbar disc herniation. Consequently, subgroup analyses specific to lumbar disc herniation remain underpowered. Secondly, **variation in rehabilitation protocols, outcome measures, and timing of follow-up** limits direct comparisons across studies and undermines the ability to draw robust conclusion regarding long-term efficacy. Additionally, although short-term benefits were evident, most studies do not examine adherence rates, postoperative continuation of rehabilitation, or cost-effectiveness, which are critical to translating findings into clinical practice.

### 4.3 Future directions

Considering the limited available data regarding physical therapy/prehabilitation in surgery in lumbar disc herniation, there appears to be a need for conducting new studies focused exclusively on this pathology and its specific characteristics. These studies should incorporate prospective, randomized controlled designs with standardized, validated outcome tools (e.g. ODI, EQ-5D, VAS, physical performance tests) and report also on adherence, patient engagement, and postoperative rehabilitation continuation. Additionally, the cost-effectiveness and scalability of different rehabilitation intensities (e.g., supervised vs. home-based) should be considered to support their integration into clinical practice.

## 5 Conclusion

While prehabilitation may provide short-term benefits in symptom relief, physical function, and psychological readiness in patients undergoing lumbar spine surgery, including those with lumbar disc herniation, the current evidence remains limited and heterogeneous. There is a clear and urgent need for well-designed prospective studies focusing on the long-term impact, optimal structure, and cost-effectiveness of prehabilitation. Addressing these gaps may support the integration of tailored prehabilitation programs into standard surgical care pathways, ultimately improving postoperative recovery, patient satisfaction, and clinical outcomes.

## Data availability statement

All data analyzed during this work is included and/or identified with proper reference in this manuscript. Further enquiries can be directed to the corresponding author.

## Statement of ethics

Ethical approval is not required for this work in accordance with the local and/or international guidelines.

## Author contributions

J. Pedro Oliveira and M. Casqueiro conducted the search in the literature and wrote the manuscript. J. Paulo Andrade also preformed the search in the literature and have contributed to revision of the manuscript. C. Reizinho reviewed, gave expert guidance and edited the manuscript.

## Declaration of generative AI and AI-assisted technologies in the writing process

The authors did not use generative AI or AI-assisted technologies in the writing process.

## Funding sources

No funding sources to report.

## Declaration of competing interest

The authors have no conflict of interest to declare.
